# Integrated bioinformatics and interaction analysis to advance chronotherapies for mental disorders

**DOI:** 10.3389/fphar.2024.1444342

**Published:** 2024-12-05

**Authors:** Apoorva Bhatnagar, Gupta Raj, Sandip Das, Arpita Kannihali, Eerappa Rajakumara, Greg Murray, Sandipan Ray

**Affiliations:** ^1^ Department of Biotechnology, Indian Institute of Technology Hyderabad, Sangareddy, Telangana, India; ^2^ Centre for Mental Health, Swinburne University of Technology, Melbourne, VIC, Australia

**Keywords:** mental disorders, chronotherapeutics, circadian rhythm, interaction analysis, molecular dynamics simulation

## Abstract

**Introduction:**

Robust connections have been identified between the pathophysiology of mental disorders and the functioning of the circadian system. The overarching objective of this study was to investigate the potential for circadian rhythms to be leveraged for therapeutics in mental disorders.

**Methods:**

We considered two approaches to chronotherapy–optimal timing of existing medications (“clocking the drugs”) and redressing circadian abnormalities with small molecules (“drugging the clock”). We assessed whether circadian rhythm-modulating compounds can interact with the prominent drug targets of mental disorders utilizing computational tools like molecular docking and molecular dynamics simulation analysis.

**Results:**

Firstly, an analysis of transcript-level rhythmic patterns in recognized drug targets for mental disorders found that 24-hour rhythmic patterns were measurable in 54.4% of targets in mice and 35.2% in humans. We also identified several drug receptors exhibiting 24-hour rhythmicity involved in critical physiological pathways for neural signaling and communication, such as neuroactive ligand-receptor interaction, calcium signaling pathway, cAMP signaling pathway, and dopaminergic and cholinergic synapses. These findings advocate that further research into the timing of drug administration in mental disorders is urgently required. We observed that many pharmacological modulators of mammalian circadian rhythms, including KL001, SR8278, SR9009, Nobiletin, and MLN4924, exhibit stable binding with psychotropic drug targets.

**Discussion:**

These findings suggest that circadian clock-modulating pharmacologically active small molecules could be investigated further for repurposing in the treatment of mood disorders. In summary, the present analyses indicate the potential of chronotherapeutic approaches to mental disorder pharmacotherapy and specify the need for future circadian rhythm-oriented clinical research.

## Introduction

**FIGURE 1:**
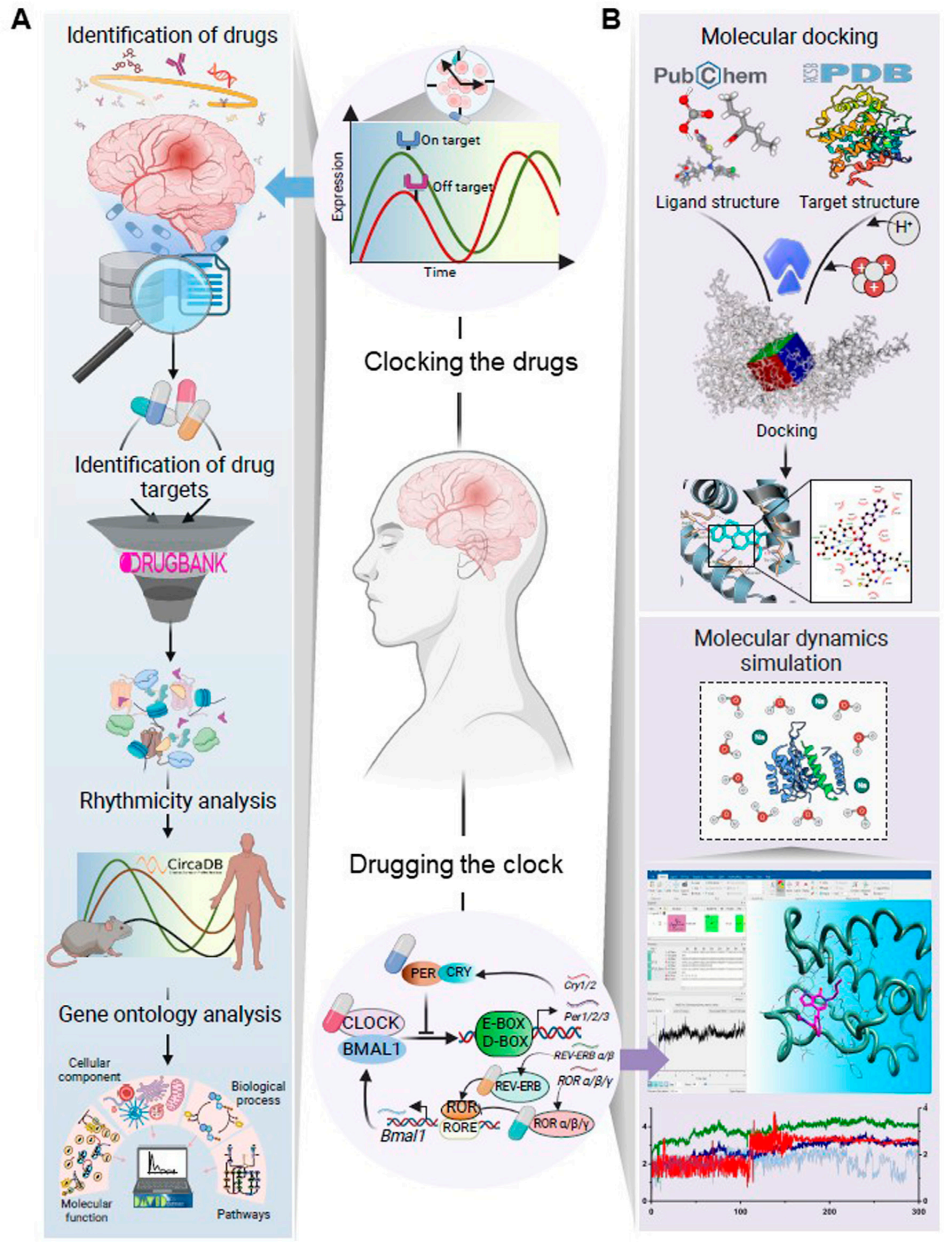
Schematic illustration of the workflow designed to investigate **(A)** the dosing time-dependency of drugs for mental disorders and **(B)** interactions of pharmacological modulators of the circadian clock with the established therapeutic targets for mental disorders. The major steps in workflow A were identifying the targets of FDA-approved medications for prevalent mental disorders, analyzing the rhythmic patterns of these targets alongside their plasma half-lives, and assessing their functional relevance through gene ontology analysis. This investigation aimed to elucidate how the rhythmicity patterns of drug targets might influence drug effectiveness, particularly in drugs with shorter plasma half-lives. Workflow B explored whether pharmacological modulators of the circadian system could interact with the known targets of psychotropic drugs using molecular docking and MD simulation analysis. This investigation aimed to assess the potential repurposing of clock-modulating pharmacologically active compounds for the treatment of mental disorders. Created with BioRender.com.

Mental disorders significantly contribute to the global disease burden, yet they remain among the most neglected areas in public health (Charlson et al., 2019). In 1990, mental disorder cases numbered around 654.8 million worldwide, increasing to 970.1 million by 2019 (GBD, 2019 Mental Disorders Collaborators, 2022). The circadian system controls human physiology and health through driving daily oscillations in a myriad of metabolic, immunological, and behavioral processes. There is growing interest in exploring how circadian rhythms can be leveraged to enhance the therapeutic management of various diseases (Giri et al., 2022; Sancar and Van Gelder, 2021; Hermida et al., 2016; Banerjee and Ray, 2023; Rankawat et al., 2023; Bhatnagar et al., 2023). Chronotherapies aim to exploit circadian rhythms to enhance drugs’ effectiveness and minimize potential adverse effects. Chronotherapeutic strategies can be categorized into three types: (1) supporting biological rhythmicity via enhancing or maintaining rhythms in feeding-fasting, sleep-wake, or light-dark, often referred to as training the clock; (2) optimizing the timing of medications according to rhythmic outputs to enhance efficiency and minimize adverse consequences, known as clocking the drugs (Ruben et al., 2019; Sulli et al., 2018a); and (3) employing small-molecule agents to target the molecular circadian clock, a strategy known as drugging the clock (Hirota et al., 2010; Lee et al., 2019; Ray et al., 2019). The present pharmacologically oriented study focuses on the latter two chrontherapeutic strategies (Figure 1).

There is evidence that the dosing time of a drug can significantly influence its efficacy. For example, an imperative study by Ruben et al. found that dosing time significantly influenced treatment outcomes in 75% of 106 clinical trials comparing treatment schedules at different times of day (Ruben et al., 2019). The aim of clocking the drugs can be achieved in a number of ways (Sulli et al., 2018a). Drug administration can be synchronized with peak expression of its physiological target or with the nadir of expression of undesirable targets (Ruben et al., 2019). Coordinating drug administration with rhythms in absorption, distribution, metabolism, or excretion (ADME) can also be beneficial (Nahmias and Androulakis, 2021). Clinical trials have shown time-dependent variations in drug action in cognition and epilepsy (Ruben et al., 2019), but there is a lack of experimental evidence on mental disorders (Bhatnagar et al., 2023). The present study sought to address this gap by systematically analyzing transcript-level rhythmicity for the established targets and receptors for commonly used drugs for various mental disorders.

Drugging the clock involves the correction of circadian dysregulation using pharmacologically active small molecules (Sulli et al., 2018a). Such small molecules interact with the central components of the molecular circadian system, allowing for the modulation of abnormal circadian period, phase, or amplitude (Bhatnagar et al., 2023; Jang et al., 2018; Miller et al., 2020). In the present study, we explored the potential binding of clock modulators with drug targets associated with mental disorders; small molecules demonstrating such binding would warrant investigation as potential chronotherapeutic drugs for mental disorders. We screened the available well-established pharmacological modulators of the molecular clock (Hirota et al., 2012; Sulli et al., 2018b; Puppala et al., 2021) for their differential interaction with receptors of some widely used medications, targeting specifically mood disorders, with the help of computational tools like molecular docking and molecular dynamics (MD) simulation. This approach can contribute to a more targeted and clinically significant understanding of the potential impact of clock modulators on mood stabilization.

## Materials and methods

Here, we introduce the three analytic steps in our Clocking the Drugs and two analytic steps in our Drugging the Clock investigations (Figure 1).

### Clocking the drugs

#### Acquisition of data regarding FDA-approved drugs, their targets, and plasma half-lives

We chose to study high-prevalence and high-burden mental disorders as indicated by the Global Burden of Disease 2019 (GBD, 2019 Mental Disorders Collaborators, 2022). We included depressive disorders, bipolar disorder, anxiety disorders, schizophrenia, conduct disorder, autism spectrum disorders, attention-deficit hyperactivity disorder, idiopathic developmental intellectual disability, and eating disorders in our study. For completeness, two prevalent neurodegenerative diseases, Parkinson’s and Alzheimer’s disease, were also included in our analyses (Lamptey et al., 2022). To compile a comprehensive list of FDA-approved medications for prominent mental disorders, we conducted searches across multiple databases (Supplementary Figure S1), including the National Institute of Mental Health (https://www.nimh.nih.gov/), the National Institute on Aging (https://www.nia.nih.gov/), and the Food and Drug Administration (FDA) (https://www.fda.gov/). Additionally, we reviewed relevant literature to further enrich our catalog of commonly used drugs for mental disorders (Ramachandraih et al., 2011; Lie et al., 2015; Greenwood et al., 2021; Davis and Attia, 2017; Christian et al., 2012). After gathering the initial list of drugs, we removed duplicate entries, resulting in a final list of 132 unique drugs for subsequent analysis. Established and potential molecular targets for these 132 drugs were fetched from a chemoinformatic resource, DrugBank v5.1.9 (https://go.drugbank.com) (Wishart et al., 2018). After removing redundant candidates, 248 molecular targets were identified for those 132 drugs (Supplementary Table S1). The plasma half-life information of these drugs, if recorded, was also retrieved from the DrugBank database (Supplementary Table S1).

#### Rhythmicity analysis of mental disorder drug targets

A rhythmicity analysis of drug targets and receptors associated with medications for mental disorders was conducted using CircaDB (http://circadb.hogeneschlab.org/). CircaDB is a database of circadian expression profiles that offers information on transcript-level oscillations across diverse tissues in both mice and humans (Pizarro et al., 2013; Ruben et al., 2018). In CircaDB, JTK_CYCLE (Hughes et al., 2010) and CYCLOPS (Anafi et al., 2017) algorithms have been used to identify the rhythmic targets in mouse and human datasets, respectively. In our analysis, we implemented a threshold of false discovery rate (FDR) of <0.1 (JTK-Q) and a circadian period of 24 ± 3 h to define rhythmicity in mice. We used FDR <0.1 and relative amplitude (rAMP) < 0.1 to detect the transcript level rhythmic candidates in human datasets (Supplementary Figure S1). We used Venny 2.1 (https://csbg.cnb.csic.es/BioinfoGP/venny.html) to analyze rhythmic targets that overlapped in mice and humans. To assess the concordance between the rhythmicity of drug targets at the transcript level and their corresponding protein expression, we compared the rhythmic transcript-level drug targets with previously published circadian proteomic data sets which report rhythmic protein expression profiles across different tissues in mammals (Robles et al., 2014; Wang et al., 2017; Chiang et al., 2014; Qian et al., 2023; Duong et al., 2024; Debski et al., 2020).

#### Identification of enriched pathways and gene ontology terms associated with rhythmic drug targets

Gene ontology (GO) and pathway analysis of rhythmic drug targets in mice (FDR <0.1) associated with mental disorders. GO analysis was conducted using the Database for Annotation, Visualization, and Integrated Discovery (DAVID) (version v2023q4) (https://david.ncifcrf.gov/) (Sherman et al., 2022). DAVID combines multiple databases (GO Biological Processes; Kyoto Encyclopedia of genes and genomes (KEGG) pathway; Reactome gene sets), providing a systematic approach to understanding the functional implications of gene sets within biological pathways. We performed an over-representation analysis using DAVID (Huang et al., 2007) to find enriched molecular functions and pathways associated with the rhythmic drug targets, where the enriched terms with FDR <0.05 were considered significant. The DAVID gene functional classification tool facilitated categorizing the rhythmic drug targets into specific GO terms, shedding light on the associated biological processes, molecular functions, and cellular components. The top five GO terms from each category and the top 20 enriched pathways were visualized using the SR plot platform (Tang et al., 2023).

#### Drugging the Clock

##### Molecular docking analysis of pharmacological modulators of the circadian clock and targets of psychotropic drugs

*In-silico* molecular docking was performed to investigate interactions between targets of psychotropic drugs and pharmacological modulators of the circadian clock using the AutoDock vina tool (version 1.1.2) (Trott and Olson, 2010). The selection of the target proteins for molecular docking analysis was guided by two criteria: firstly, they had to be receptors for essential psychotropic medicines employed in the treatment of mental disorders, and secondly, they needed to exhibit prominent circadian expression patterns characterized by circadian period of 24 ± 3 h and a minimum JTK Q-value. Based on the above-mentioned selection criteria, the following eight target proteins were chosen for analysis, GSK3β (PDB ID: 5K5N; targeted by lithium), MTNR1A (PDB ID: 6ME3; targeted by Melatonin), CALM1 (PDB ID: 4BW8; targeted by Melatonin), CALR (PDB ID: 3POW; targeted by Melatonin), PPARα (PDB ID: 1I7G; targeted by Valproic acid), ADRB1 (PDB ID: 7BVQ; targeted by aripiprazole), SIGMAR1 (PDB ID: 5HK1; targeted by Haloperidol), ACHE (PDB ID: 4MOE; targeted by Donepezil) were identified for molecular docking analysis.

Circadian system-modulating pharmacologically active small molecules targeting the core and adjacent clock components, including cryptochrome (CRY), nuclear receptor subfamily one group D member 1 (REV-ERB), ROR, and glycogen synthase kinase three beta (GSK3β), were systematically searched and selected for molecular docking analysis as ligands. Ligands’ 3D structures were obtained from PubChem (Kim et al., 2022) and converted from SDF to PDB files using PyMOL (http://www.pymol.org) (PyMOL, 2024). The three-dimensional structures of drug-receptor proteins were sourced from the Protein Data Bank (https://www.rcsb.org/) (Berman et al., 2000). Docking was conducted five times for each ligand, and the standard error of the mean binding free energy (BE) was evaluated. The best docking pose was determined by selecting the conformation with the lowest BE (kcal/mol).

We used an established interaction between valproic acid (VPA) - peroxisome proliferator-activated receptor alpha (PPARα) as a positive control for evaluating interactions of pharmacological modulators of the circadian clock with the targets of the drugs for mental disorders. VPA is routinely used in managing epilepsy and mood disorders and has been identified as an agonist of PPARα (Szalowska et al., 2014). VPA is also a circadian rhythm modulator that enhances circadian amplitude and changes phase (Johansson et al., 2011).

##### Molecular dynamics simulation analysis

In the MD simulation analysis, a focused approach was taken by selecting specific drug targets and pharmacological modulators of circadian rhythms with known associations with mood stabilization or clinical relevance. To this end, among the eight drug targets analyzed by molecular docking, GSK3β, PPARα, and Melatonin receptor type 1A (MTNR1A) were chosen for MD simulation analysis. GSK3β and PPARα are linked to commonly prescribed mood stabilizers, namely, lithium and VPA, respectively. MTNR1A is a cell membrane receptor for the hormone melatonin, which can potentially ameliorate sleep and circadian components of mental disorders (Moon et al., 2022). Among the recognized clock modulators, we selected five for MD simulation analysis - SR9009, SR8278, Nobiletin, KL001, and MLN4924 as they are currently being investigated for their mood-stabilizing properties.

MD simulations of GSK3β, PPARα, and MTNR1A docked with each ligand were carried out using the Desmond engine (Desmond Molecular Dynamics System, Version 2.2, D.E. Shaw Research, New York, NY, 2009). The complexes were solvated in water using the TIP3Pew model with 0.15 M NaCl, neutralized with expected counter ions (Na + or Cl-), and enclosed within an orthorhombic box with dimensions of 10 in each direction and orthogonal angles (α = β = γ = 90^o^). The systems were reduced in size and set to Desmond’s standard operating procedures. The OPLS3e force field was employed to govern the system’s dynamics, and long-range electrostatic interactions were computed using the particle-mesh Ewald method (Darden et al., 1993). A cut-off radius of 9.0 was employed to consider van der Waals and Coulomb interactions at short ranges. The system’s pressure and temperature were maintained via the Martyna-Tobias-Klein method (Martyna et al., 1994) and the Nose-Hoover thermostat (Hoover, 1985), respectively. A consistent time step of 2 fs was applied for the entire simulation. Subsequently, each system underwent an individual 200–500 ns MD simulation. The preservation of metal coordination geometry during simulations was ensured by establishing zero-order connections to metal ions using the Protein Preparation Wizard panel in Maestro and Schrodinger (PyMOL, 2024). The PRODIGY-LIGAND web server (Vangone et al., 2019) was employed to compute the ΔG values of complexes post-MD simulations.

## Results

### A significant proportion of the targets for mental disorder drugs display robust daily rhythms

**FIGURE 2:**
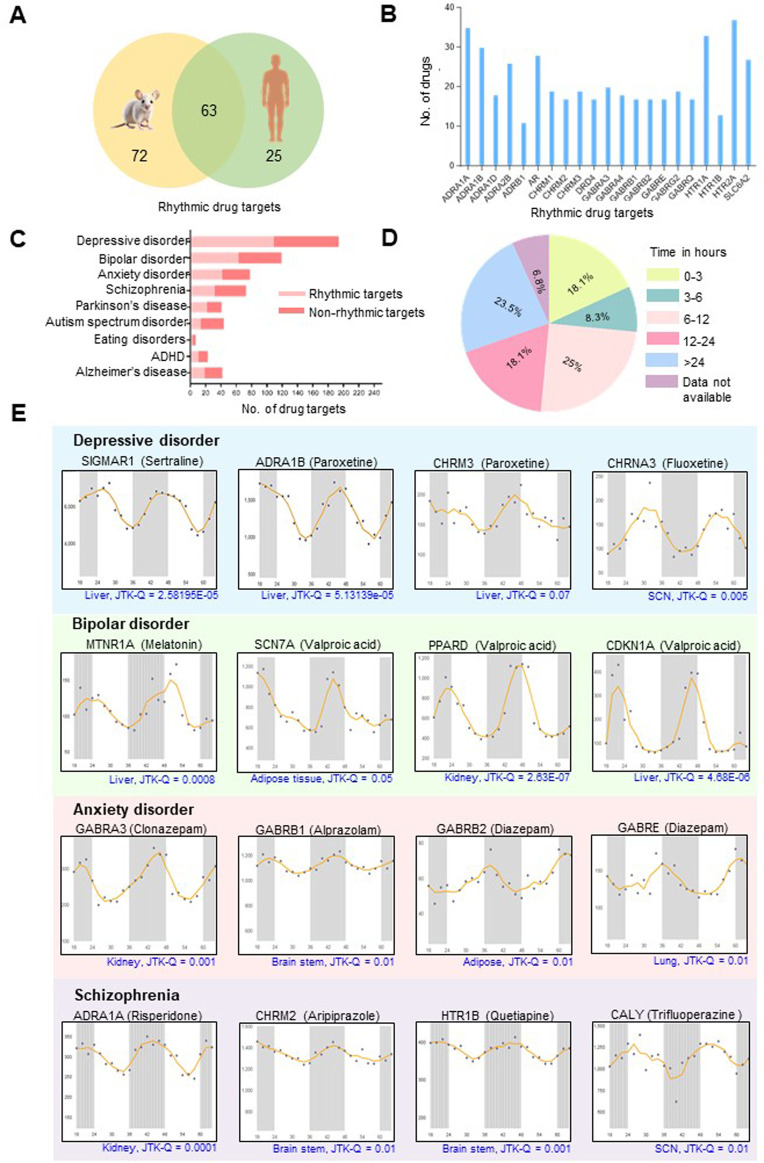
Molecular targets for mental disorder drugs display robust daily rhythms. **(A)** Venn diagram showing overlapping rhythmic targets of drugs routinely used for mental and neurological disorders in mice and humans (Period 24 ± 3 h, JTK Q < 0.1). Information regarding the transcript-level rhythmic expression patterns of the targets is obtained from the CircaDB database (http://circadb.hogeneschlab.org/). **(B)** Multiple drugs share common 24-h rhythmic drug targets. **(C)** Distribution of rhythmic drug targets in different mental and neurological disorders. **(D)** Pie chart illustrating the distribution of plasma half-lives for mental disorder drugs with rhythmic targets. Drugs with plasma half-lives of less than 15 h and robustly rhythmic targets are most likely influenced by the dosing time (See Supplementary Table S1 for details). **(E)** Transcript-level rhythmic (Period±24 h, JTK Q < 0.1) expression patterns of representative drug targets for the four most prevalent mental disorders. The *X*-axis indicates time (h), and the *Y*-axis designates normalized intensity.

Searching for the drug targets for 132 mental disorder drugs revealed that many drugs share common cellular targets (Supplementary Table S1). A total of 248 molecular drug targets were analyzed for possible rhythmic patterns using the CircaDB platform. Amongst these, we identified 135 (54.4%) and 88 (35.4%) rhythmic candidates (Period 24 ± 3 h, JTK Q < 0.1) in mice and humans, respectively (Supplementary Table S2). A comparative analysis of rhythmic drug target genes in mice and humans revealed 63 rhythmic overlapping candidates (Figure 2A). Among 135 rhythmic drug targets found in mice, it was observed that multiple drugs share several molecular rhythmic targets. We identified 21 rhythmic drug targets that interact with 10 or more different drugs (Figure 2B). Notably, HTR2A is a common target for 37 different drugs, and 35 drugs target ADRA1A (Figure 2B). Furthermore, several drug targets, including HTR1A (33 drugs), AR (28 drugs), and SLC6A2 (27 drugs), are targeted by multiple drugs. The existence of overlapping molecular targets across multiple drugs highlights the possibilities of shared molecular pathways or mechanisms across existing treatments for mental disorders. Figure 2C illustrates the prevalence and distribution of rhythmic drug targets among various mental disorders. We observed that a considerable proportion of drug targets for prominent mental disorders show robust 24-h rhythmicity, such as 57% for depressive disorder (110 out of 194), 54% for bipolar disorder (64 out of 119), 54% for anxiety disorders (42 out of 78) and 42% for schizophrenia (30 out of 71) (Figure 2C). A comparative analysis of the transcriptomics and proteomics data sets indicated that 37 targets for mental disorder drugs exhibit rhythmicity at both protein and transcript levels (Supplementary Table S2). The protein level expression data on a circadian time scale is unavailable for many targets for mental disorder drugs in the existing literature.

Per the study conducted by Ruben et al., 85% of trials involving drugs with half-lives less than 15 h exhibited dosing time dependence (Ruben et al., 2019). In our study, an evaluation of the plasma half-lives for the mental disorder drugs with 24-h rhythmic targets or interactors revealed that over 50% exhibit half-lives of less than 15 h (Figure 2D; Supplementary Table S1). We also found that 18.1% of these drugs have half-lives shorter than 3 h (Figure 2D). Tuning the time of administration of mental disorder drugs with narrow half-lives and robust rhythmic targets will be essential.

### Targets of mental disorder drugs show temporal variations across multiple organs

The CircaDB analysis revealed targets of mental disorder drugs have 24-h transcript-level rhythmic profiles across various organs, including the brain, cerebellum, brain stem, liver, kidney and suprachiasmatic nucleus (SCN the ‘master clock’) (Figure 2E). Rhythmic drug targets such as CHRNA3 and CALY in the SCN, along with GABRB1, CHRM2, and HTR1B in the brain stem, highlight the possible circadian regulation of neurotherapeutic and neurobiological processes influencing mental health disorders and their treatment. Besides the brain, rhythmicity of transcripts in peripheral organs, such as the liver and kidneys, is essential to consider while targeting mental diseases. These organs control drug metabolism and excretion, influencing their pharmacokinetics. Understanding these rhythms may aid in optimizing drug dosing times for better efficacy and reduced side effects. Additionally, mental disorders often have systemic effects and comorbid medical conditions affecting other systems (Wang Z. et al., 2022), so studying how drug targets function in peripheral tissues is important in understanding the overall impact of treatments. The 24-h rhythmic patterns were observed for several drug targets in the liver, such as MTNR1A and CALM1, suggest that the liver’s responsiveness to melatonin and its ability to synthesize dopamine may fluctuate throughout the day. Additionally, rhythmic targets in the kidney, like ADRA1A, which impact renal blood flow and filtration rate, offer insights into the circadian regulation of renal functions and drug responsiveness.

Our analysis revealed that multiple targets for the same medication, such as targets of VPA (SCN7A, PPARD, and CDKN1A) and the anxiolytic drug Diazepam targets (GABRB2 and GABRE), show similar temporal patterns across the day (Figure 2E). Recognizing these synchronized rhythmic patterns in drug targets across various organs can inform the development of tailored treatment strategies. This understanding can lead to more personalized and effective therapeutic approaches, enhancing treatment efficacy and pharmacokinetic predictability.

### Rhythmic targets within biological pathways have functional implications

**FIGURE 3:**
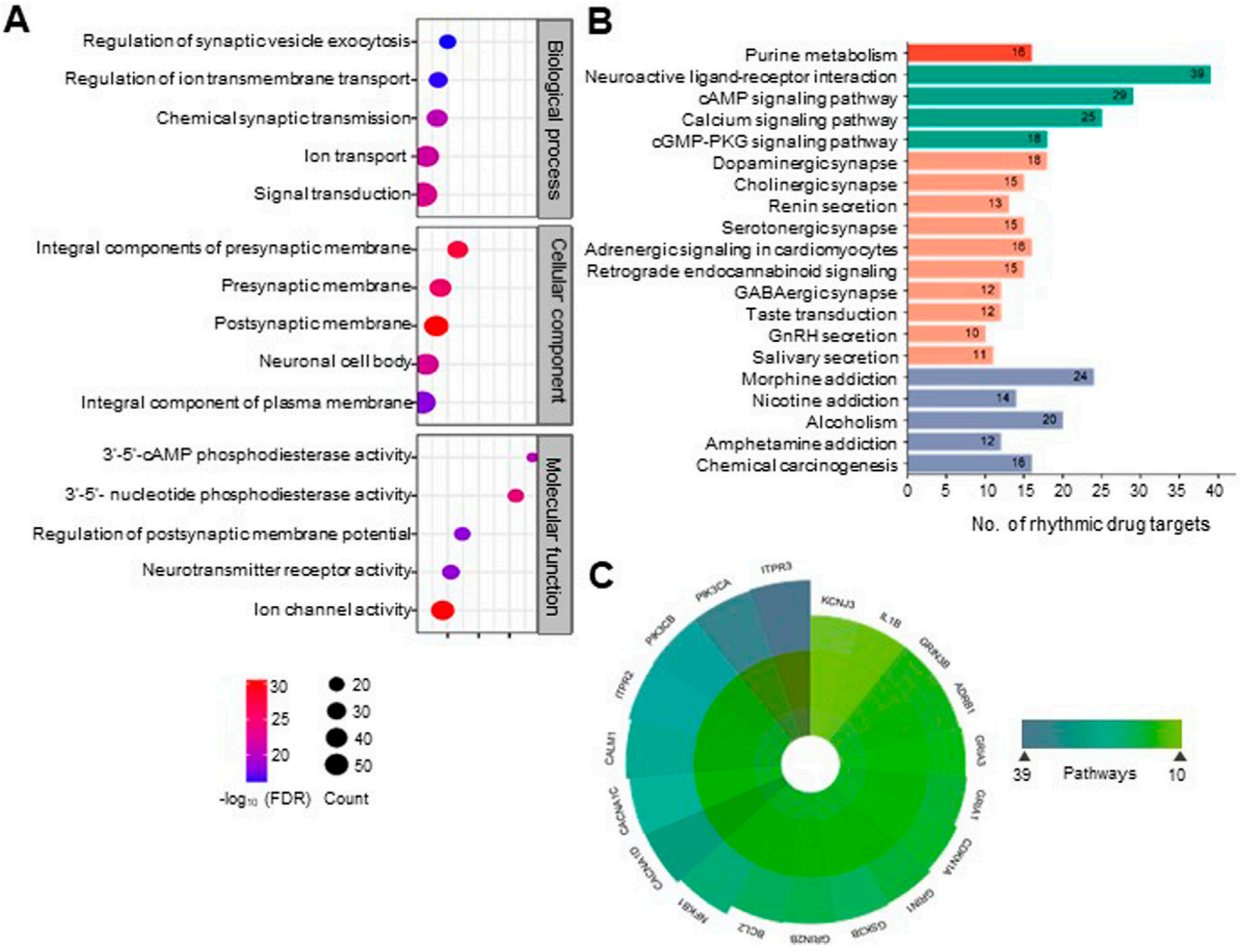
Rhythmic targets for mental disorder drugs are associated with diverse essential neurobiological processes and physiological pathways. **(A)** Categorizing rhythmic drug targets into functional groups by integrating information from the DAVID database (https://david.ncifcrf.gov/). Overrepresented biological processes, molecular functions, and cellular components are defined for the rhythmic drug targets (FDR <0.1). The top five statistically enriched (FDR <0.01, enrichment factor >1.5, count >5) GO terms from each category are depicted (See Supplementary Table S3–5 for details). **(B)** Physiological pathways associated with the molecular targets of mental disorder drugs. Pathways were obtained from the DAVID database (KEGG pathways). The top 20 statistically enriched (FDR <0.05, enrichment factor >1.5, count >5) canonical pathways are shown (see Supplementary Table S6 for details). **(C)** We observed that many rhythmic drug targets are associated with multiple physiological pathways. A polar plot depicted the highly widespread rhythmic drug targets involved in more than ten physiological pathways.

GO analysis of rhythmic drug targets indicated that many therapeutic targets for mental disorders are involved in signal transduction and ion transport across synapses (Figure 3A; Supplementary Tables S3–5). This finding aligns with the involvement of rhythmic drug targets in critical pathways related to neural signaling and communication. Notably, the majority of these rhythmic drug targets are prominently associated with pathways such as neuroactive ligand-receptor interaction (Q = 1.11E-20, fold enrichment (FE) = 7.42), cyclic AMP signaling pathway (Q = 1.87E-17, FE = 9.54) calcium signaling pathway (Q = 3.45E-12, FE = 7.24), dopaminergic synapse (Q = 3.18E-11, FE = 10.18), and cholinergic synapse (Q = 2.45E-09, FE = 10.23) (Figure 3B; Supplementary Table S6). Among these associated pathways, the neuroactive ligand-receptor interaction pathway has the maximum number of rhythmic targets, 38 in count (Figure 3B). In the context of mental disorders, these pathways play crucial roles in neurotransmission, synaptic signaling, and neuronal communication, all of which are integral to regulating mood, cognition, and emotional wellbeing.

**FIGURE 4:**
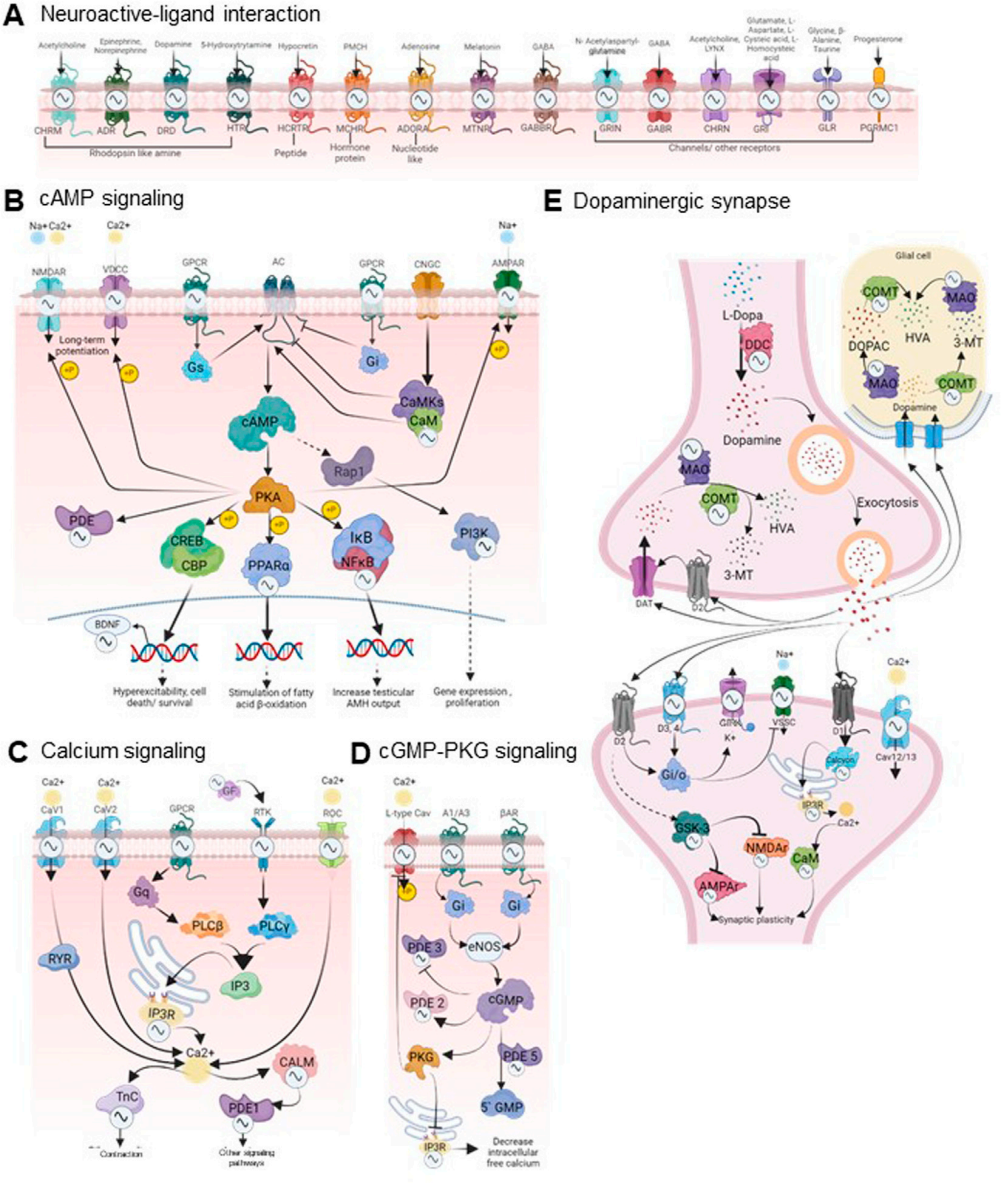
Circadian regulation of drug response in mental disorders. The figure visually represents the circadian regulations of the components of five key signaling pathways of the brain that are frequently targeted by the majority of mental disorder drugs (FDR <0.05, fold enrichment >2.5, count >5). The rhythmicity symbols specify the transcript level rhythmicity (JTK Q < 0.1, period 24 ± 3 h) of that component in the particular signaling pathway. **(A)**The neuroactive ligand-receptor interaction pathway encompasses G protein-coupled receptors (GPCRs) for neurotransmitters such as dopamine, serotonin, GABA, and acetylcholine, along with downstream effectors like calcium ion channels, cAMP, cGMP, PKA, and PKG. The downstream signaling cascades are displayed in panels **(B–E)**, which play crucial roles in the pathophysiology of various psychiatric disorders by affecting gene expression, neuronal excitability, responses, and synaptic plasticity (Perez and Tardito, 2001; Reierson et al., 2011; Nakao et al., 2021). These pathways are closely intertwined and form a complex signaling network. A partial view of these interconnected pathways encompassing several rhythmic drug targets is depicted here. The complete pathway diagrams with rhythmic drug targets are shown in Supplementary Figure S5. Signaling pathways impacted by mental disorder drugs were retrieved from the DAVID database (https://david.ncifcrf.gov/). The rhythmicity of each component involved was investigated using the CircaDB database (http://circadb.hogeneschlab.org/). Created with BioRender.com.

We observed that a large number of rhythmic drug targets involved in signaling pathways are located on both pre and post-synaptic membranes of neurons and are involved in phosphodiesterase activity (3′-5′-cAMP and 3′-5′- nucleotide phosphodiesterase activity) affecting neurotransmission (Figure 3A). Figure 3C highlights specific targets’ multifaceted roles across various biological processes by prioritizing drug targets with 24-h rhythmic transcripts that are involved in at least ten pathways. Notably, ITPR3 and PIK3CA are involved in 39 and 38 pathways, respectively, suggesting biological pathways are interconnected and functionally related, leading to coordinated changes in complex neurobiological processes. This interconnectedness allows for a more holistic approach to understanding how the circadian regulation of a drug can exert broader effects on various biological processes and can help identify circadian time-oriented effective therapeutic strategies. In Figure 4, we visually depicted the five key pathways significantly influenced by drugs for mental disorders and their 24-h rhythmic components.

### Pharmacological modulators of the circadian system exhibit high affinity towards the established therapeutic targets of mental disorders

The predicted BE for all drug targets with their respective ligands (Supplementary Table S7) obtained in molecular docking analysis are presented in Supplementary Table S8. Notably, the majority of clock modulators showed a considerable binding affinity for mental disorder drug targets, with consistently lower BE (Range: 5.4 to −11.7 kcal/mol) compared to the positive control VPA-PPARα interaction (−9.04 kcal/mol). For instance, interactions between SIGMAR1 and KL001, PPARα and CHIR99021 displayed a BE of −9.04 kcal/mol. SIGMAR1- MLN4924 interaction displayed a BE of −11.06 kcal/mol, while ACHE-LY2090314 binding showed a BE of −11.7 kcal/mol. Supplementary Figure S2 depicts the comparison of BE for all targets, emphasizing that lower BE may correspond to more stable binding interactions.

**FIGURE 5:**
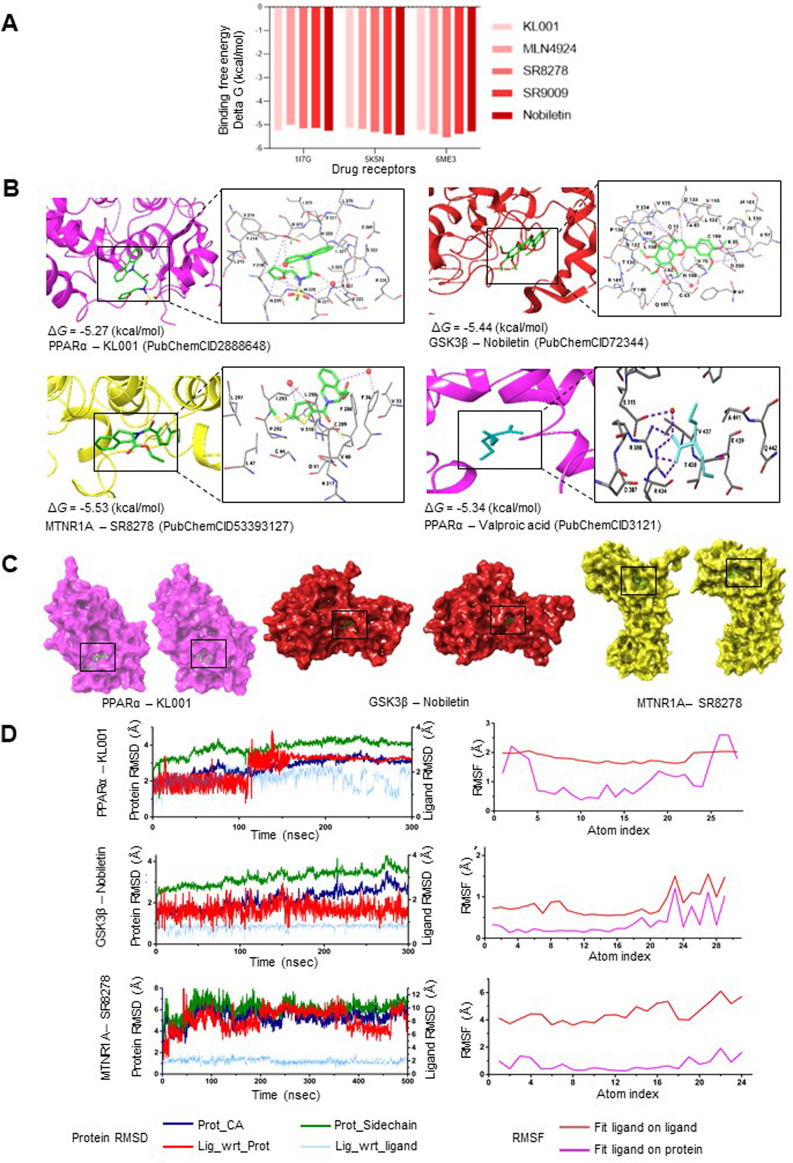
Stable interactions between the pharmacological modulators of the circadian system and the established therapeutic targets of mental disorders **(A)** Binding free energy comparison of the 3D structure of pharmacological circadian modulators used for MD simulation analysis with molecular targets of lithium (GSK3β; PDB ID: 5K5N), valproic acid (PPARα; PDB ID: 1I7G), and melatonin (MTNR1A; PDB ID: 6ME3). **(B)** Binding models and interaction sites of PPARα, GSK3β, and MTNR1A with clock modulators exhibiting best binding affinity and stabilization within the receptor binding pocket (See Supplementary Table S10 for details). **(C)** Snapshots are shown at the beginning (left) and end (right) of the simulation for the best interaction models among PPARα, GSK3β, and MTNR1A with their ligands (clock modulators). **(D)** Root Mean Square Deviation (RMSD) and Root Mean Square Fluctuation (RMSF) values for the most robust interactions (PPARα-KL001, GSK3β-Nobiletin, and MTNR1A-SR8278) are displayed as a function of simulation time.

MD simulations were performed to evaluate the stability of ligand-protein complexes. Figure 5A represents the BE computed using the MD-simulated ligand-protein complexes after attaining stable conformations. KL001, Nobiletin, and SR8278 exhibited slightly higher ΔG_bind_ for 1I7G (−5.27 kcal/mol), 5K5N (−5.44 kcal/mol), and 6ME3 (−5.53 kcal/mol), respectively, as compared to other ligands. Figure 5B shows the interaction profiles of PPARα-KL001, GSK3β-Nobiletin, and MTNR1A-SR9278 complexes. The snapshots of complexes (protein represented as surface) at the beginning and the end of the simulation are shown in Figure 5C. Binding model interactions of PPARα, GSK3β, and MTNR1A with remaining circadian clock modulators are depicted in Supplementary Figure S3. Minimal fluctuations in the root mean square deviation (RMSD) of protein and ligand and root mean square fluctuation (RMSF) of the ligand indicated complex stability during the simulation (Figure 5D; Supplementary Figure S4). VPA exhibited a ΔG_bind_ of −5.34 kcal/mol with 1I7G, serving as a positive control for comparing the BE of all simulated structures, revealing comparable results with all the simulated structures (Figure 5B). Stable binding poses were observed for most of the ligands on various targets after specific simulation durations.

We observed that pharmacological modulators of the clock protein REV-ERBs (SR9009 and SR8278) exhibit robust interaction with psychotropic drug targets. SR9009 achieved stable binding with PPARα (1I7G), MTNR1A (6ME3), and GSK3β (5K5N) at 200ns, 150ns, and 180ns, respectively. In 200–300ns MD simulated protein-ligand complexes, SR9009 attained stabilizing interactions and conformations via water-mediated H-bonds with specific residues in 6ME3 (D124, L127, F65, I151) (Supplementary Figure S4). SR8278 attained a stable binding pose with PPARα, MTNR1A, and GSK3β after 250ns, 470ns, and 120ns, respectively.

Similarly, Nobiletin, a clock amplitude-enhancing small molecule, exhibited a stable binding pose with PPARα, MTNR1A, and GSK3β after 270ns, 220ns, and 200ns, respectively. A 300ns MD simulated protein-ligand complex revealed that Nobiletin made stabilizing interactions via water-mediated H-bonds and direct H-bonds with particular residues in 5K5N (N186, Y140, D200, D133, V135, Y134) (Figure 5B). MLN4924, which stabilizes the retinoid orphan nuclear receptor alpha (RORα), exhibited stabilizing pi-interactions H-bonds and water-mediated H-bonds in 6ME3 (V124, Q216, G1001, L213, L229, S1194, D1003) (Supplementary Figure S4). KL001, which interacts with the core clock proteins CRY1 and CRY2, also reached stability with PPARα, MTNR1A, and GSK3β after 180ns, 120ns, and 300ns, respectively.

## Discussion

The findings of the present study suggest that the recognized potential of chronotherapies for a range of health conditions extends to common, highly burdensome mental health disorders. The circadian system regulates mood, cognitive functions, and a range of neurobiological processes. Aligning drug administration with circadian rhythms has the potential to enhance treatment efficacy while minimizing toxic side effects, as demonstrated by the rhythmicity of drug targets identified in the present analyses. Multiple transcriptome studies have shown robust 24-h rhythmicity of thousands of protein-coding genes in mammals in a tissue-specific manner, many of which are involved in the drug ADME processes (Ruben et al., 2018; Zhang et al., 2014; Mure et al., 2018). Circadian rhythms also profoundly influence drugs’ pharmacokinetics and pharmacodynamics by orchestrating temporal variations in drug receptors and their target pathways (Nahmias and Androulakis, 2021). Rhythmic expressions of the primary targets for drugs with short half-lives emphasize the need for precise drug administration timing to align with their respective receptors’ peak expression. For example, Buspirone, used for anxiety disorders, has a brief half-life of roughly 2 h. Administering Buspirone during the circadian peak of its receptors activity, including DRD2 and DRD3 (Le Foll et al., 2016) may augment its anxiolytic effects. Similarly, Zolpidem, a sedative-hypnotic with an elimination half-life of about 2.5 h, might be more effective when given during the circadian window of heightened sensitivity to sedation. Tailoring the timing of drug administration according to circadian rhythms can optimize therapeutic outcomes and minimize potential side effects associated with the medications commonly prescribed for mental disorders.

Some psychotropic drugs can have multiple physiological targets and interactors that exhibit 24-h rhythmicity. Variability in acrophases among multiple targets of such drugs could be a critical consideration in chronotherapy. Multiple targets of a particular drug can exhibit the expression peak at similar or adjacent timings, such as SCN7A, PPARD, and CDKN1A (targets for VPA) or GABRB2 and GABRE (targets for Diazepam) show almost comparable acrophases (Figure 2E). However, for drugs with multiple targets having substantial differences in the acrophases, determining the optimal timing for drug administration based only on the expression peak of its targets would be difficult. Consequently, under those circumstances, the rhythmic peak of the most therapeutically relevant target as well as rhythmicity in other ADME processes, may need to be considered. While the rhythmicity of the physiological targets can provide an important clue regarding the likely dosing-time dependency of the pharmaceutical agents, evaluating their time-dependent variations in therapeutic efficacy (and toxicity) in pre-clinical models and clinical setups is paramount for establishing their chronotherapeutics applicability.

The complicated interplay between circadian rhythms and neurobiological processes, including neurotransmitter, hormonal regulation, and sleep-wake cycles, emphasizes the importances of temporal alignment in therapeutic interventions. Our findings highlighed that the primary focus of drugs targeting mental disorders includes the calcium signaling pathway, cAMP signaling pathway, cGMP-PKG signaling pathway, and dopaminergic synapse. We observed that several elements within these signaling pathways are under circadian regulation, as evidenced by their rhythmic expression at the transcript level (Figure 4; Supplementary Figure S5). Rhythmic drug targets within these pathways imply a potential circadian influence on neurobiological processes relevant to mental health. cAMP and cGMP play pivotal roles in regulating mental disorders through the phosphorylation of cAMP response element-binding protein (CREB) (Delhaye and Bardoni, 2021; Wang S. et al., 2022; Dwivedi and Pandey, 2008). Under stress conditions, elevated CREB levels reduce sensitivity to emotional and stress stimuli (Barrot et al., 2002). Antipsychotics boost CREB activity, contributing to the recovery of disrupted sensorimotor gating seen in schizophrenia and various mood disorders (Culm et al., 2004). The present analyses show that multiple components of the cAMP and cGMP-PKG signaling pathway targeted by several psychotropic drugs display 24-h rhythms in mice and humans. Given the rhythmic nature of these drug receptors, optimizing drug administration to align with their circadian patterns can enhance efficacy.

Drug repurposing offers a pragmatic and efficient approach to drug development, capitalizing on the wealth of knowledge available for existing drugs and potentially bringing new treatment options to patients more rapidly and at a lower cost (Pushpakom et al., 2019; Lüscher Dias et al., 2020). Our molecular docking results indicated favorable binding interactions between pharmaceutical agents that modulate the circadian system and mood stabilizer receptors. MD simulations provided further understanding by assessing the stability of predicted binding poses over time. Given the known impact of pharmacological compounds on circadian rhythms, their interaction with receptors related to mental disorders presents a dual benefit. By modulating these receptors, circadian system manipulation may regulate neurobiological processes linked to mental health. This understanding lays the groundwork for investigating the repurposing of circadian clock modulators as potential therapeutic agents for mental health conditions, effectively drugging the clock to address psychiatric disorders.

Another critical aspect in exploring circadian-targeting therapeutics for mood disorders is investigating the possibilities of direct binding of psychotropic drugs to the core components of the circadian system. For instance, lithium, a commonly used mood stabilizer, has been shown to directly inhibit glycogen synthase kinase three beta (GSK3β), a vital clock-controlling kinase. Lithium’s inhibition of GSK3β leads to stabilizing circadian rhythms, which may also contribute to its mood-stabilizing effects in bipolar disorder (Yin et al., 2006). *In vivo* studies have shown that lithium leads to circadian period elongation in peripheral clock gene expression (Sawai et al., 2019). However, whether lithium’s effects on the peripheral clock gene expression is through direct binding with any core clock proteins is not established. Therefore, investigating the possibilities of direct interactions between psychotropic drugs and the core components of the circadian system will be an imperative extension of our current study.

While our *in silico* analysis (clocking the drugs) provided insights into potential rhythmic drug targets, caution is warranted in interpreting these findings. A time delay exists between RNA transcription and protein translation, leading to a phase difference of rhythmicity between the transcript and protein levels. Also, rhythmicity observed at the transcriptional level may not always match temporal patterns at the protein level, leading to a relatively low correlation between rhythmic transcriptome and proteome (Schenk et al., 2019; Ray et al., 2020). Post-translational modifications can further influence 24-h rhythmicity patterns. Rhythmic patterns may also differ across cell types and tissues, emphasizing the need for cell and tissue-specific considerations in rhythmicity analyses. Integrating multi-omics circadian data, including transcriptomics, proteomics, and metabolomics, is essential for a comprehensive understanding of the molecular rhythmicity of mental disorder drug targets and associated neurobiological processes. More importantly, large-scale clinical trials will be obligatory to validate the dosing time dependency of such mood disorder medications.

Our drugging the clock evaluation provided preliminary insights into binding affinities and structural dynamics, offering a groundwork for further examination. However, the inherent limitations of *in silico* analyses demand thorough validation in *in-vitro* or *in-vivo* settings before considering clinical application. Biological systems are dynamic, context-dependent, and subject to systemic responses, environmental influences, and adaptability over time. *In-silico* analyses conducted here may not fully capture these dynamic changes, potentially leading to disparities between predictions and real-world outcomes. Additionally, the models may lack a comprehensive representation of pharmacokinetic properties and potential toxicity concerns, which are crucial considerations in the translation of drug candidates. Given these limitations, *in-vitro* and *in-vivo* validation is imperative to ensure the reliability and relevance of our findings in a clinical context.

## Conclusion

We propose that the dearth of evidence-driven chronotherapeutic approaches is a missed opportunity for pharmacological treatment of mental disorders. The present study demonstrates that rigorous methods can illuminate opportunities for optimizing the therapeutic timing of existing mental health drugs (clocking the drug) and repurposing small molecules to stabilize molecular clock function (drugging the clock). The pathway from laboratory to clinic is long, but the present findings give us confidence that efforts in this direction are warranted: Mental disorders are complex systemic conditions, and adaptive circadian timing appears to be a key modifiable feature of this complexity.

## Data Availability

The original contributions presented in the study are included in the article/Supplementary Material, further inquiries can be directed to the corresponding authors.
