# Supplementary material for: Integrated bioinformatics and interaction analysis to advance chronotherapies for mental disorders
Source: Front Pharmacol. 2024 Dec 5;15:1444342. doi: 10.3389/fphar.2024.1444342 (PMC11655208; doi:10.3389/fphar.2024.1444342)
Supplement: Supplementary file 4 [file DataSheet1.pdf]

Supplementary Material

Research article

Integrated bioinformatics and interaction analysis to advance chronotherapies for mental disorder

Apoorva Bhatnagar[1, 2], Gupta Raj[1], Sandip Das[1], Arpita Kannihali[1], Eerappa Rajakumara [1], Greg Murray[2]*, Sandipan Ray[1]*

[1]Department of Biotechnology, Indian Institute of Technology Hyderabad, Kandi, Sangareddy, 502284, Telangana, India

[2]Centre for Mental Health, Swinburne University of Technology, Melbourne, Victoria, Australia

*Correspondence: sandipan.ray@bt.iith.ac.in (S. Ray); gwm@swin.edu.au (G. Murray)

List of supplementary figures:

Figure S1. Flowchart showing the analysis pipeline for investigating rhythmicity of drug targets of mental disorder drugs and analyzing the interaction between the drug targets of mental disorder drugs and pharmacological modulators of the circadian system with mood stabilization effects.

Figure S2. Average binding free energy comparison of all the molecular drug receptors used for the docking analysis with circadian clock modulators as ligands.

Figure S3. Binding model interactions of PPARα, GSK3β, and MTNR1A with remaining circadian clock modulators.

Figure S4. Fluctuation in PPARα, GSK3β, and MTNR1A structure with remaining clock modulators during the MD simulation. RMSD for all the chains of proteins as a function of simulation time and RMSF per residue averaged over the simulation time are displayed.

Figure S5. The five major signaling pathways in the brain, primarily targeted by the majority of mental disorder drugs (FDR < 0.05, fold enrichment > 2.5, count > 5), were identified. Rhythmicity symbols indicate the transcript-level rhythmicity (JTK Q < 0.1, period 24 ± 3 hours) of each component within these pathways. Components marked with the day and night-shaded rhythmicity symbols represent targets of mental disorder drugs under circadian control. Pink-colored rhythmicity symbols signify pathway components controlled by the circadian clock but are not directly targeted by mental disorder drugs.

List of supplementary tables:

Table S1. Details of the molecular targets for FDA-approved routinely used mental disorder drugs. (In separate Excel file)

Table S2. Rhythmic expression of mental disorder drug targets in mammalian systems. (In separate Excel file)

Table S3. Summary of the cellular components associated with the rhythmic drug targets. (In separate Excel file)

Table S4. Summary of the molecular functions associated with the rhythmic drug targets. (In separate Excel file)

Table S5. Summary of the biological processes associated with the rhythmic drug targets. (In separate Excel file)

Table S6. Summary of the pathways associated with the rhythmic drug targets. (In separate Excel file)

Table S7. Details of the ligands used in docking analysis. (In separate Excel file)

Table S8. Binding free energies of the interaction studies performed by autodocking (N=5). (In separate Excel file)

Table S9. Details of ligands used in MD simulation.

Table S10. Binding free energies of interaction studies by MD simulation analysis.

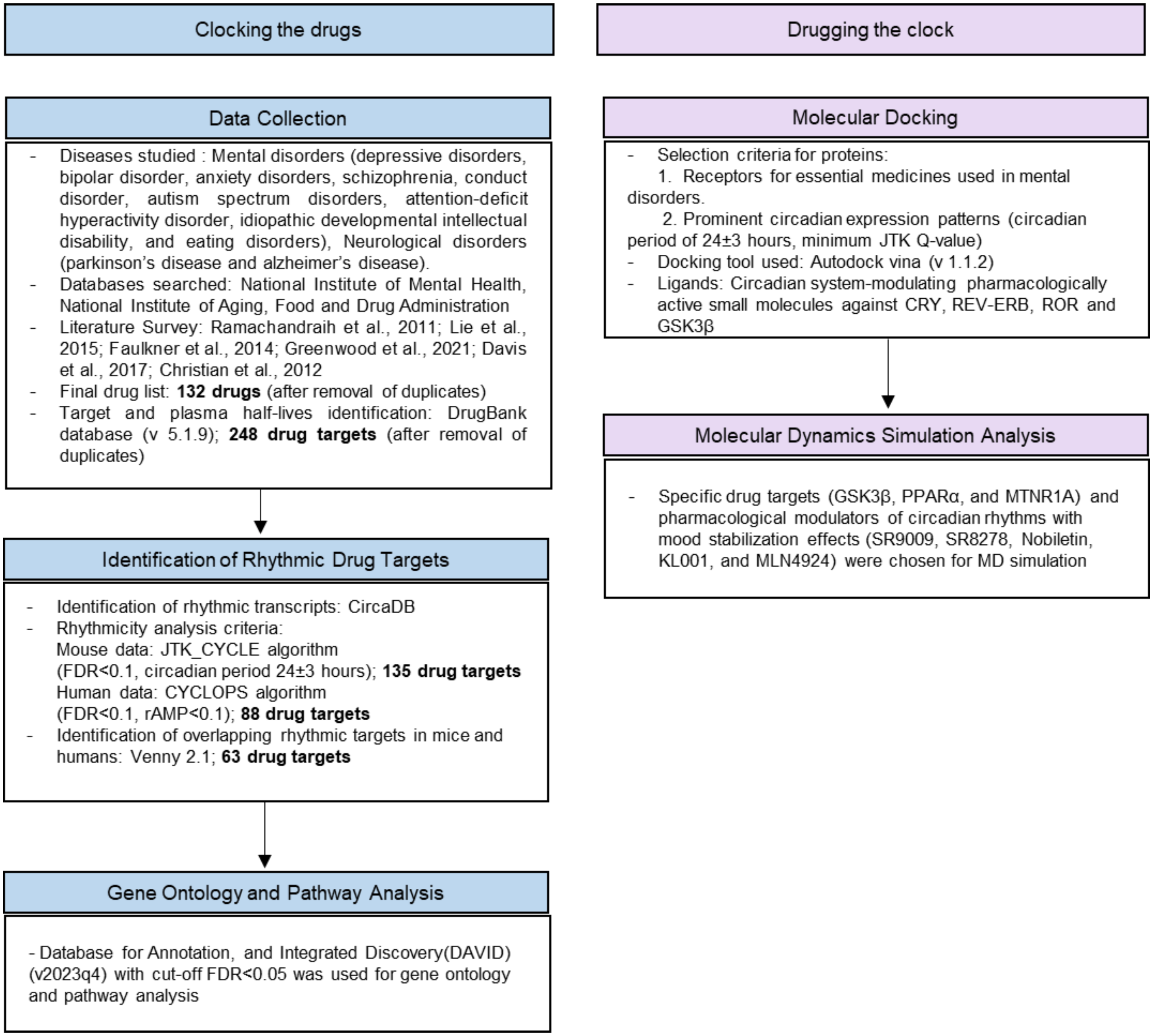

Figure S1. Flowchart showing the analysis pipeline for investigating rhythmicity of drug targets of mental disorder drugs and analyzing the interaction between the drug targets of mental disorder drugs and pharmacological modulators of the circadian system with mood stabilization effects.

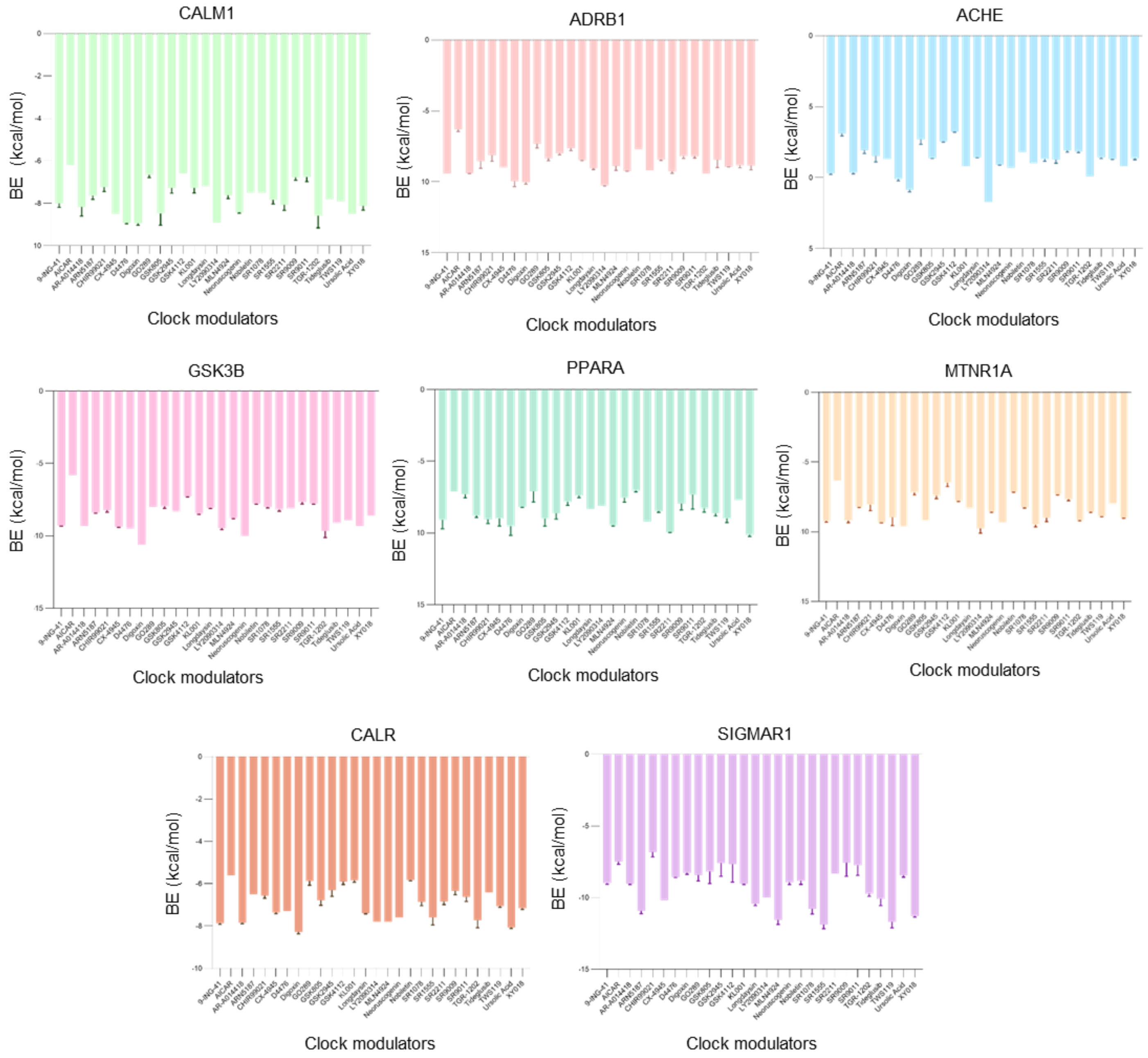

Figure S2. Average binding free energy (BE) comparison of all the molecular drug receptors used for the docking analysis with circadian clock modulators as ligands.

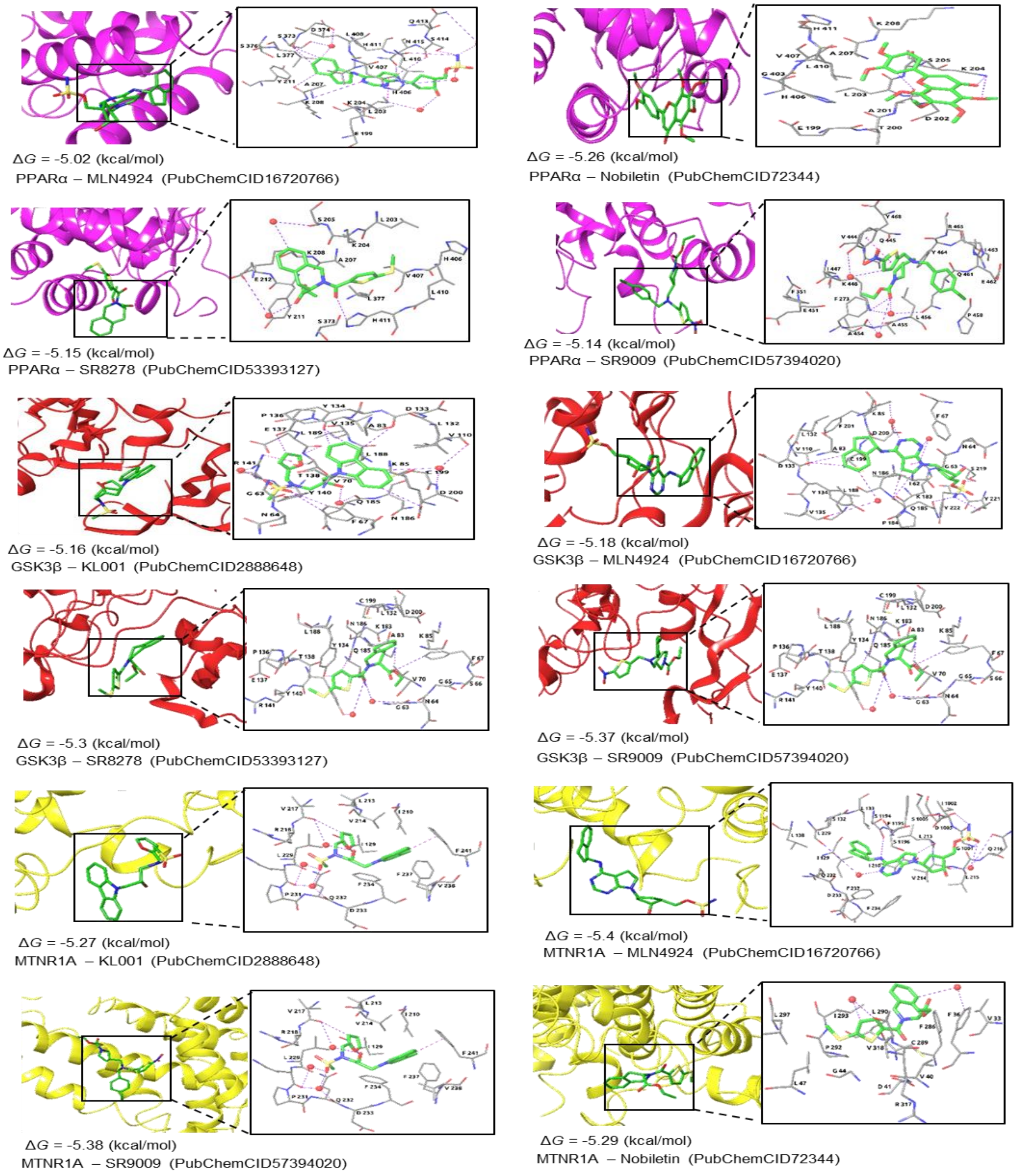

Figure S3. Binding model interactions of PPARα, GSK3β, and MTNR1A with remaining circadian clock modulators.

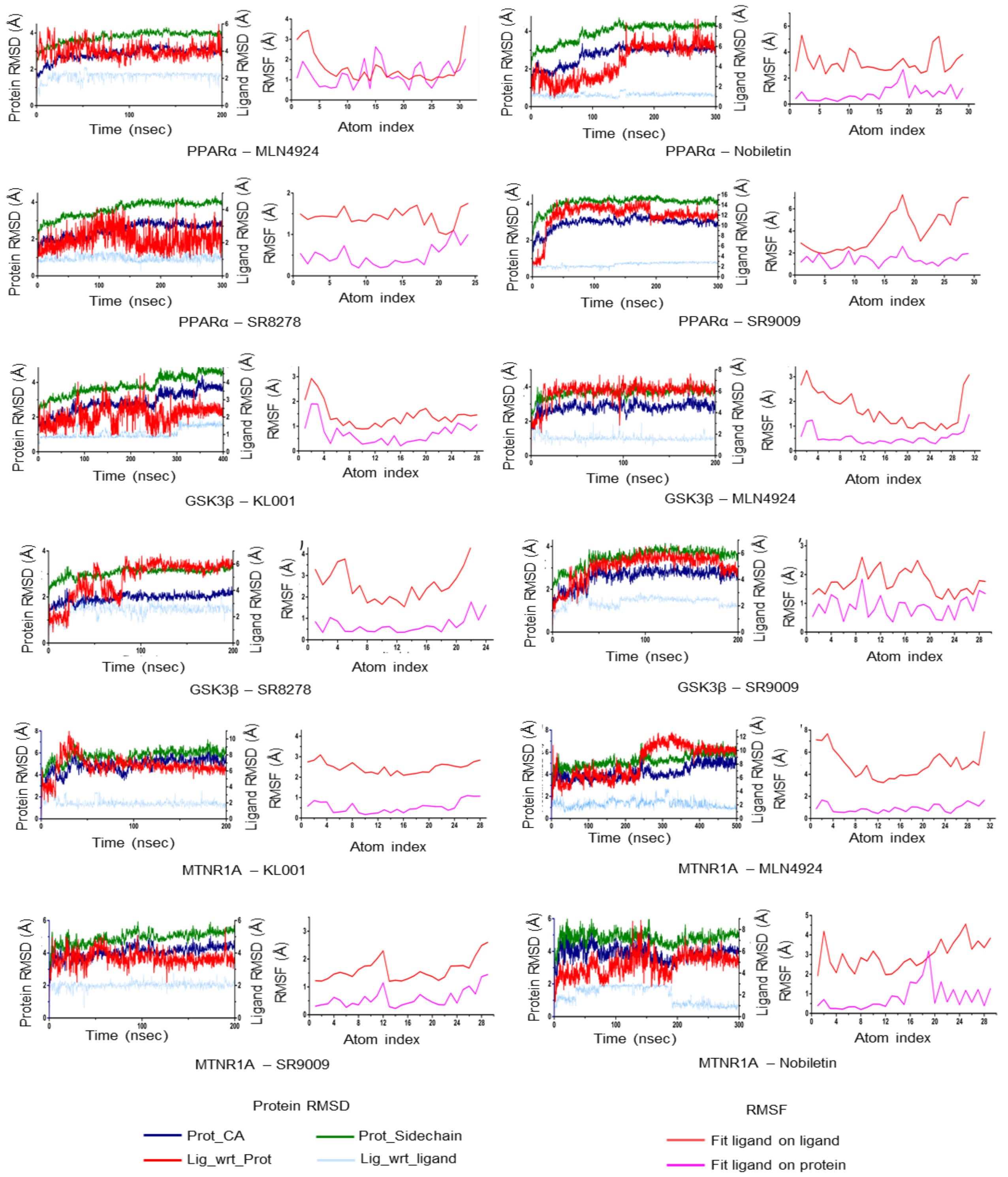

Figure S4. Fluctuation in PPARα, GSK3β, and MTNR1A structure with remaining clock modulators during the MD simulation. RMSD for all the chains of proteins as a function of simulation time and RMSF per residue averaged over the simulation time are displayed.

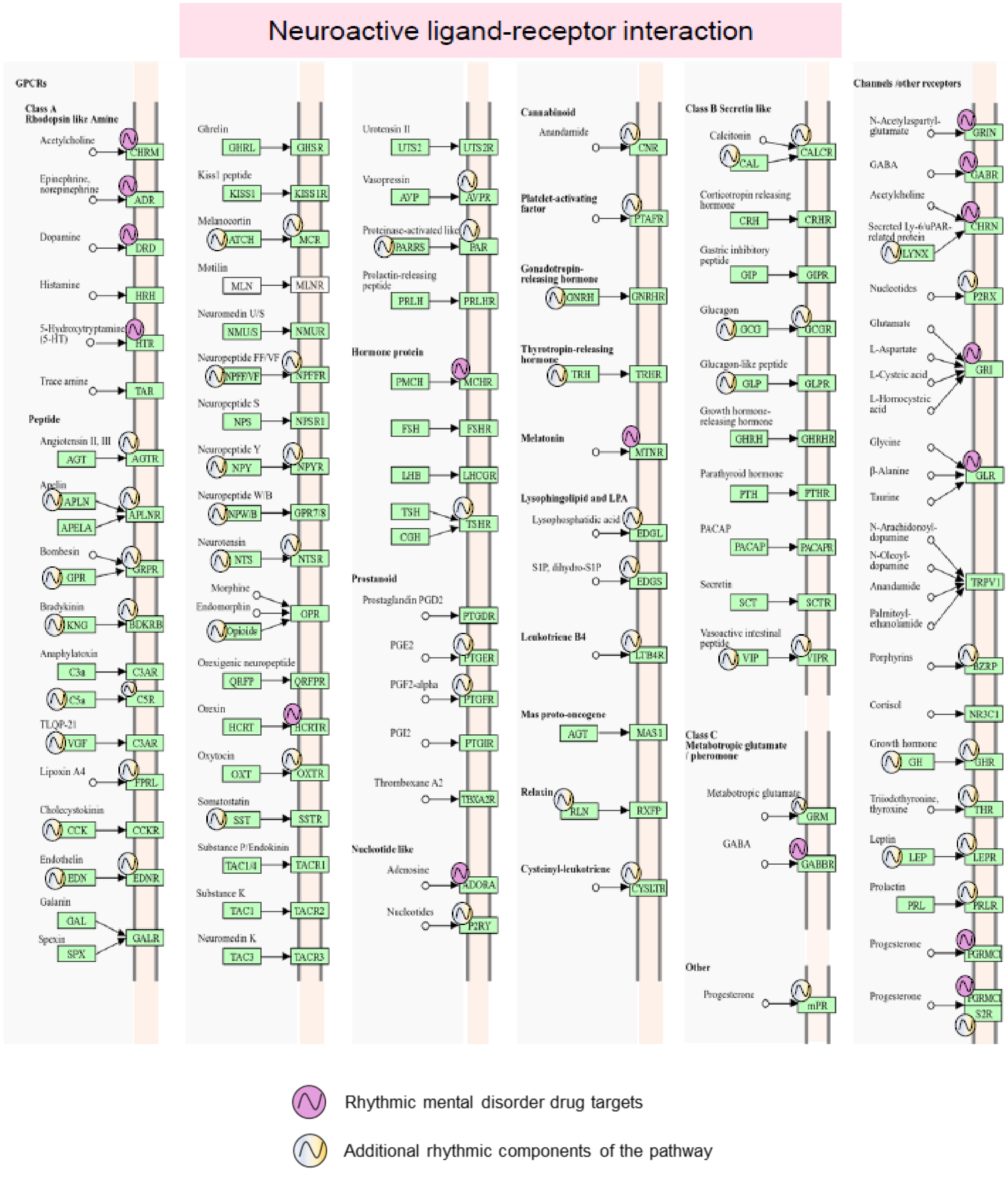

Neuroactive ligand-receptor interaction
GPCRs
Class A
Rhodopsin like Amine
Acetylcholine
CHRM
Epinephrine, norepinephrine
ADR
Dopamine
DRD
Histamine
HRH
5-Hydroxytryptamine (5-HT)
HTR
Trace amine
TAR
Peptide
Angiotensin II, III
AGT
AGTR
Apelin
APLN
APELA
APLNR
Bombesin
GPR
GRPR
Bradykinin
KNG
BDKRB
Anaphylatoxin
C3a
C3AR
C5a
C5R
TLQP-21
VGF
C3AR
Lipoxin A4
FPRL
Cholecystokinin
CCK
CCKR
Endothelin
EDN
EDNR
Galanin
GAL
Spexin
SPX
GALR
Ghrelin
GHRL
GHSR
Kiss1 peptide
KISS1
KISS1R
Melanocortin
ATCH
MCR
Motilin
MLN
MLNR
Neuromedin U/S
NMU/S
NMUR
Neuropeptide FF/VF
NPFF/VF
NPFFR
Neuropeptide S
NPS
NPSR1
Neuropeptide Y
NPY
NPYR
Neuropeptide W/B
NPW/B
GPR7/8
Neurotensin
NTS
NTSR
Morphine
Endomorphin
Opioids
OPR
Orexigenic neuropeptide
QRFP
QRFPR
Orexin
HCRT
HCRTR
Oxytocin
OXT
OXTR
Somatostatin
SST
SSTR
Substance P/Endokinin
TAC1/4
TACR1
Substance K
TAC1
TACR2
Neuromedin K
TAC3
TACR3
Urotensin II
UTS2
UTS2R
Vasopressin
AVP
AVPR
Proteinase-activated like
PARRS
PAR
Prolactin-releasing peptide
PRLH
PRLHR
Hormone protein
PMCH
MCHR
FSH
FSHR
LHB
LHCGR
TSH
CGH
TSHR
Prostanoid
Prostaglandin PGD2
PTGDR
PGE2
PTGER
PGF2-alpha
PTGFR
PGI2
PTGIR
Thromboxane A2
TBXA2R
Nucleotide like
Adenosine
ADORA
Nucleotides
P2RY
Cannabinoid
Anandamide
CNR
Platelet-activating factor
PTAFR
Gonadotropin-releasing hormone
GNRH
GNRHR
Thyrotropin-releasing hormone
TRH
TRHR
Melatonin
MTNR
Lysophingolipid and LPA
Lysophosphatidic acid
EDGL
S1P, dihydro-S1P
EDGS
Leukotriene B4
LTB4R
Mas proto-oncogene
AGT
MAS1
Relaxin
RLN
RXFP
Cysteinyl-leukotriene
CYSLTR
Class B Secretin like
Calcitonin
CAL
CALCR
Corticotropin releasing hormone
CRH
CRHR
Gastric inhibitory peptide
GIP
GIPR
Glucagon
GCG
GCGR
Glucagon-like peptide
GLP
GLPR
Growth hormone-releasing hormone
GHRH
GHRHR
Parathyroid hormone
PTH
PTHR
PACAP
PACAP
PACAPR
Secretin
SCT
SCTR
Vasoactive intestinal peptide
VIP
VIPR
Class C
Metabotropic glutamate / pheromone
Metabotropic glutamate
GRM
GABA
GABBR
Other
Progesterone
mPR
Channels /other receptors
N-Acetylaspartyl-glutamate
GRIN
GABA
GABR
Acetylcholine
Secreted Ly-6/uPAR-related protein
LYNX
CHRN
Nucleotides
P2RX
Glutamate
L-Aspartate
L-Cysteic acid
L-Homocysteic acid
GRI
Glycine
β-Alanine
Taurine
GLR
N-Arachidonoyl-dopamine
N-Oleoyl-dopamine
Anandamide
Palmitoyl-ethanolamide
TRPV1
Porphyrins
BZRP
Cortisol
NR3C1
Growth hormone
GH
GHR
Triiodothyronine, thyroxine
THR
Leptin
LEP
LEPR
Prolactin
PRL
PRLR
Progesterone
PGRMC1
Progesterone
PGRMC1
S2R
Rhythmic mental disorder drug targets
Additional rhythmic components of the pathway

cGMP- PKG signaling pathway

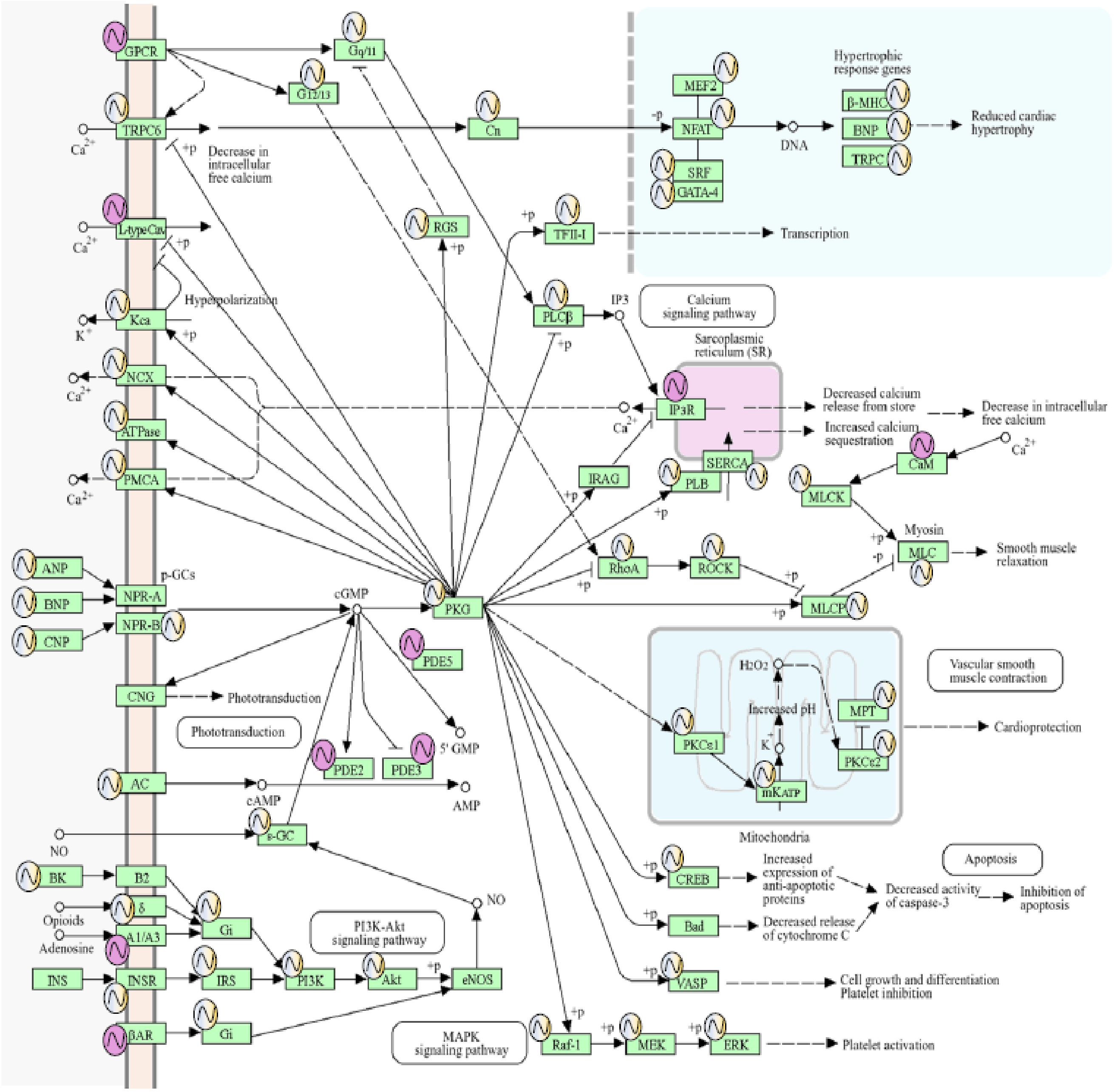

Rhythmic mental disorder drug targets

Additional rhythmic components of the pathway

cAMP signaling pathway

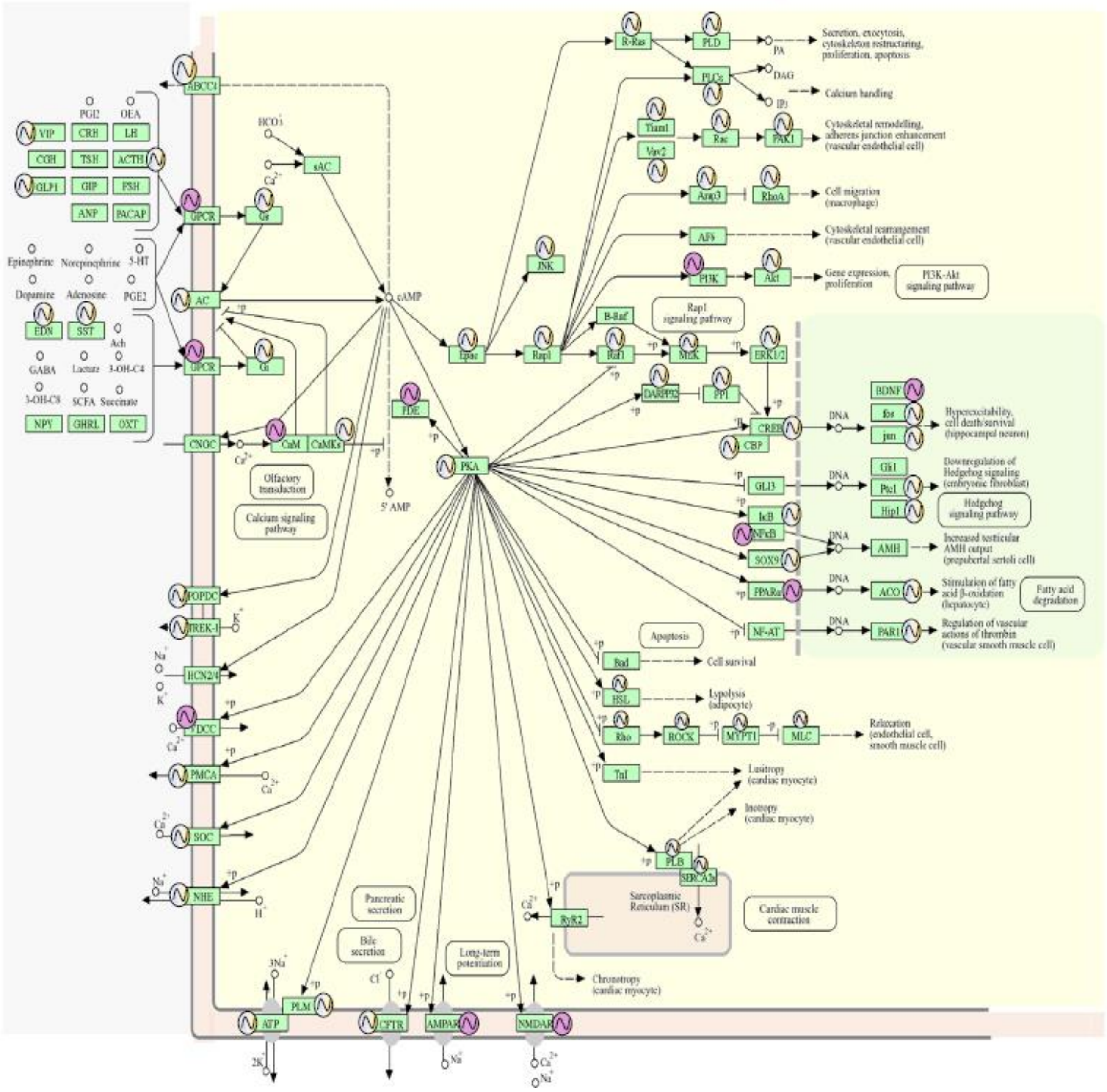

Rhythmic mental disorder drug targets

Additional rhythmic components of the pathway

Calcium signaling pathway

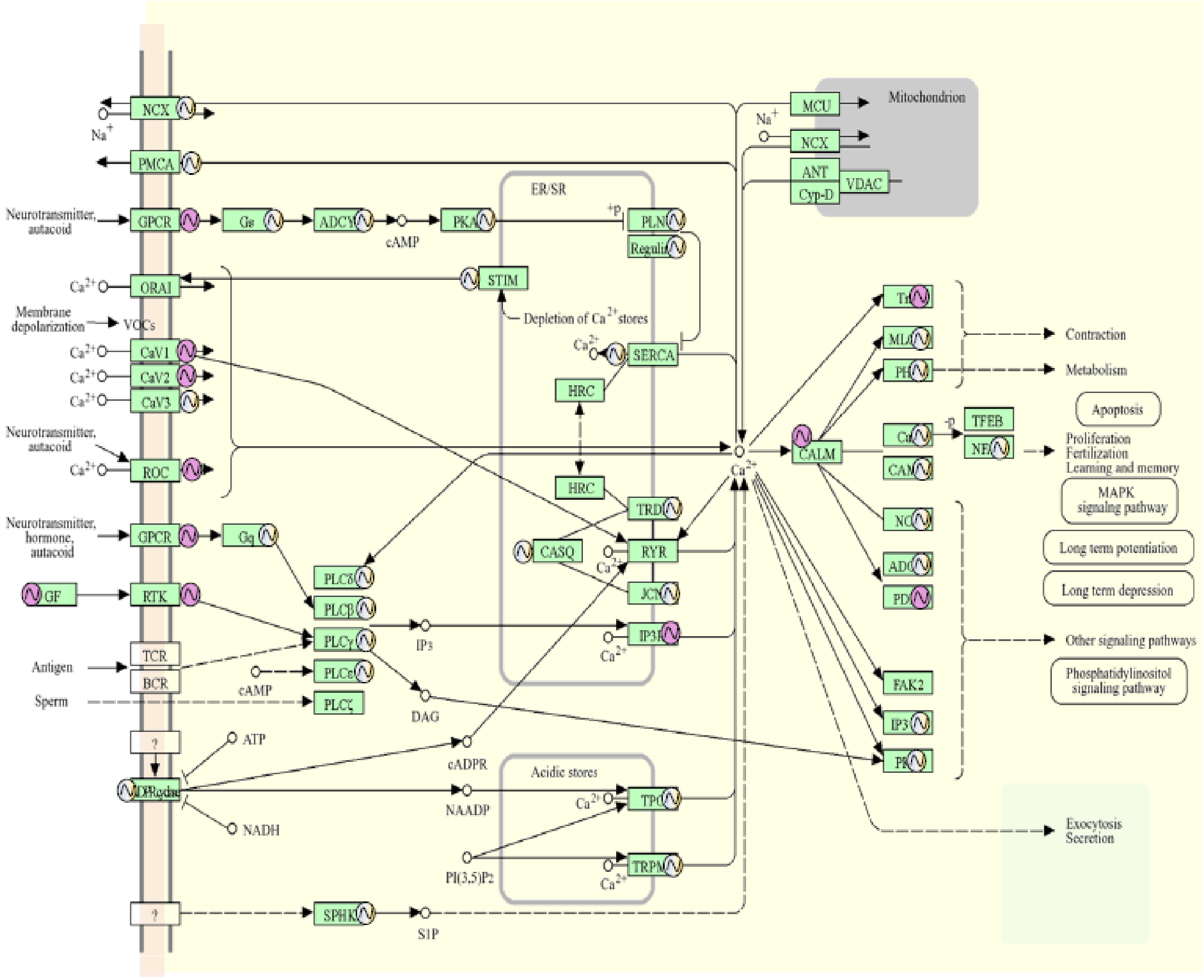

Rhythmic mental disorder drug targets

Additional rhythmic components of the pathway

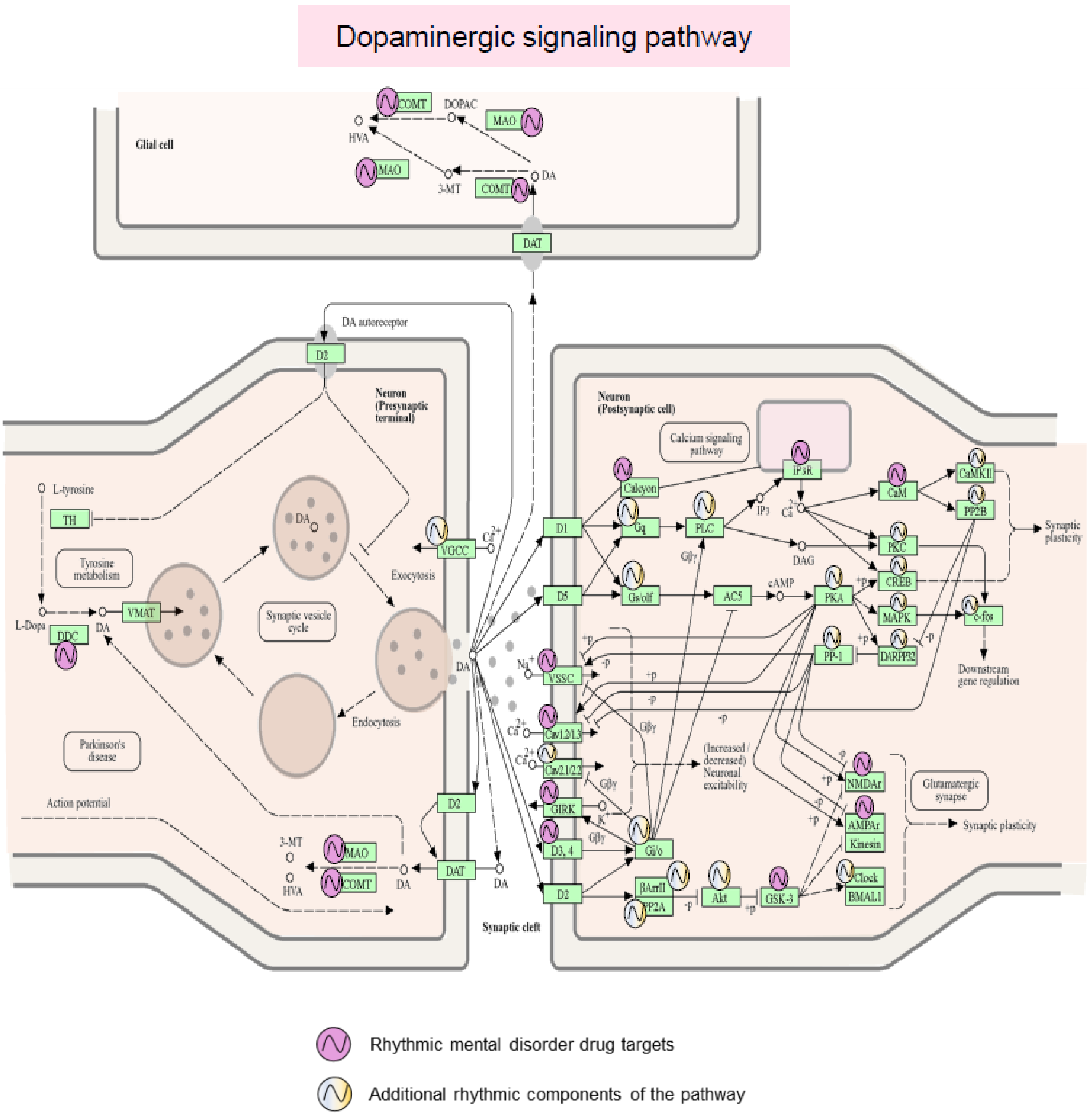

Figure S5. The five major signaling pathways in the brain, primarily targeted by the majority of mental disorder drugs (FDR < 0.05, fold enrichment > 2.5, count > 5), were identified. Rhythmicity symbols indicate the transcript-level rhythmicity (JTK Q < 0.1, period 24±3 hours) of each component within these pathways. Components marked with pink-colored symbols represent targets of mental disorder drugs that are under circadian control. Day and night-shaded rhythmicity symbols signify pathway components that are controlled by the circadian clock but are not direct targets of the mental disorder drugs.

Table S9. Details of ligands used in MD simulation

S. No.	Ligand	Clock component	Mode of action	Effect on circadian clock assessed	Reference	Effect on mood assessed	Clinical trial
1	SR9009	REV-ERB α/β	REV-ERB agonist	Alter circadian pattern of expression of core clock genes	Solt et al., 2012, PMID: 22460951	Anxiolytic effect Banerjee et al., 2014, PMID: 25536025	No
2	SR8278	REV-ERB α/β	REV-ERB antagonist	Increases Bmal1 transcription	Kojetin et al., 2011, PMID: 21043485	Reduces depressive like behaviour Chung et al., 2014, PMID: 24813609	No
3	Nobiletin	RORs	ROR α/γ agonist	Enhances the amplitude of the circadian rhythm of PER2::LUC	Ambe et al., 2022, PMID: 36431828	Anti-depressant Yi et al., 2011, PMID: 20951716	No
4	KL001	CRY 1/2	CRY1 activator	Lengthens the circadian period	Solovev et al., 2022, PMID: 34449576	No	No
5	MLN4924	RORs	ROR-α stabilization	Upregulates the expression of BMAL1	Zhang et al., 2016, PMID: 27602774	No	Yes, Phase 2 completed for AML, Active not recruiting for multiple cancers

Table S10. Binding free energies of interaction studies by MD simulation analysis

	Ligands				
**Protein**	KL001	MLN4924	SR8278	SR9009	Nobiletin
**1I7G**	-5.27	-5.02	-5.15	-5.14	-5.26
**5K5N**	-5.16	-5.18	-5.3	-5.37	-5.44
**6ME3**	-5.27	-5.4	-5.53	-5.38	-5.29
